# Longitudinal Task-Related Functional Connectivity Changes Predict Reading Development

**DOI:** 10.3389/fpsyg.2018.01754

**Published:** 2018-09-19

**Authors:** Gregory J. Smith, James R. Booth, Chris McNorgan

**Affiliations:** ^1^Department of Psychology, State University of New York at Buffalo, Buffalo, NY, United States; ^2^Department of Psychology and Human Development, Vanderbilt University, Nashville, TN, United States

**Keywords:** functional connectivity, functional magnetic resonance imaging (fMRI), graph theory, reading network, reading development, longitudinal, neural connectivity

## Abstract

Longitudinal studies suggest developmentally dependent changes in lexical processing during reading development, implying a change in inter-regional functional connectivity over this period. The current study used functional magnetic resonance imaging (fMRI) to explore developmental changes in functional connectivity across multiple runs of a rhyming judgment task in young readers (8–14 years) over an average 2.5-year span. Changes in functional segregation are correlated with and predict changes in the skill with which typically developing children learn to apply the alphabetic principle, as measured by pseudoword decoding. This indicates a developmental shift in the proportion of specialized functional clusters is associated with changes in reading skill and suggests a dependency of reading development on changes of particular neural pathways, specifically decreases in transitivity is indicative of greater network integration. This work provides evidence that characteristics of these pathways, quantified using graph-theoretic metrics, can be used to predict individual differences in reading development.

## Introduction

Reading is a multisensory task requiring audiovisual integration of printed characters and speech sounds. This entails coordinated contributions of multiple functionally specialized brain regions implicated in reading, coined the *reading network*, including the ventral occipito-temporal cortex (vOT), middle and superior temporal gyri (MTG, STG), inferior frontal gyrus (IFG), angular gyrus (AG), and inferior parietal lobule (IPL), connected by arcuate fasciculus (AF), inferior fronto-occipital fasciculus (IFOF), and other dense white-matter tracts that facilitate communication among these regions (Paus et al., 1999; Pugh et al., 2001; Marchina et al., 2011). Analysis of blood-oxygen-level dependent (BOLD) signal in developing readers shows changes in cortically distributed processes at these regions over time predict changes in reading fluency (McNorgan et al., 2011). Together, these findings suggest an important role of connectivity in reading performance that presumably entails changes in interregional connectivity.

A body of research provides evidence for developmentally dependent diffuse structural and functional changes in processing within the reading network for developing readers. Notably, considerable research provides evidence for a developmental shift from phonological to orthographic processing in developing readers (Sprenger-Charolles et al., 2003; Booth et al., 2007; Blomert, 2011). Reading fluency is dependent on changing anatomical connectivity between some of these regions: diffusion tensor imaging (DTI) of white matter tract passing through core multisensory hub (AF) predicted cortical activity during an audiovisual phonological judgment task (Gullick and Booth, 2014), with the direct segment of AF predicting longitudinal changes in reading development (Gullick and Booth, 2015). This shift is presumably accompanied by changes in how regions supporting phonological and orthographic processing communicate with each other during reading. Indeed, this is supported by recent research using a rhyming judgment task that shows a decrease in dorsal stream connectivity, responsible for phonological processing, is important for the emergence of ventral stream dependent orthographic processing (Younger et al., 2017). Comparison of task-related functional connectivity show disparate regional activation for children and adults, with children showing greater temporal functionality associated with phonological processing, and adults a greater task-related sensitivity with reliance on occipital regions associated with orthographic processing (Liu et al., 2018), which provides convergent functional evidence in favor of developmentally dependent changes across the reading network.

Connectivity studies further show that the nature of the developmental changes to reading network are related to reading outcomes. Greater dorsal striatum connectivity is associated with poorer adult reading performance, as measured using a pseudoword decoding task, which may reflect inefficient lexical processing (Achal et al., 2016). This is consistent with research showing less efficient processing (e.g., a high degree of integrated functional connectivity across a more diffuse network) within regions associated with a narrative comprehension task for children with reading difficulties compared to task-dependent regions of normal readers (Horowitz-Kraus et al., 2016). Whole-brain functional connectivity analysis for English as a second language (ESL) individuals indicates more localized clusters for an English phonological rhyming task in second language reading impaired children compared to normal controls (Liu et al., 2016). In an analysis of a functionally defined reading network, similar to that in the present study, Wang et al. (2013) found that greater interaction between distal hierarchically segregated network clusters is associated with better rhyming judgment task performance. Together, these results suggest that language processing benefits from cooperative integrative processing among processing centers in the reading network.

Collectively, these findings suggest improvements in reading skill are predicted by the nature and degree of changes among connectivity patterns within the reading network and are consistent with general theories of neural development that implicate synaptic pruning and myelination as a mechanism that optimizes processing efficiency by reducing noise believed associated with excess connectivity (Huttenlocher, 1979; Tau and Peterson, 2010; Navlakha et al., 2015). Not all cognitive processes are likely to depend equally on the same structural connections between cortical and subcortical regions; however, changes in anatomical connectivity should nonetheless be reflected in changes in functional connectivity, which describes patterns of interregional signaling during a functional MRI task. The present study capitalizes on this dynamic relationship between structural and functional connectivity by exploring how measures of task-related functional connectivity explain individual differences in reading ability, and thus suggest critical connectivity-related mechanisms on which reading development relies.

Our longitudinal study aims to identify the changes in patterns of functional connectivity among core nodes in the reading network that are predictive of developmental changes in single word reading skill. The literature discussed above suggests several potential connectivity-dependent mechanisms within the reading network that are predictive of reading skill. In isolation, each of these findings provide insight into the dependence of reading on the development of particular constituents of this network. Though the existing literature clearly shows that connectivity strength among some of these regions is an important determinant of reading skill, we use graph-theoretic metrics that quantify how, rather than how much, the global reading network is connected, to test the hypothesis that reading skill depends on the degree to which these anatomically distributed and functionally specialized processing centers work in concert. Single word reading skill was assessed for the analyses that follow using the Test of Word Reading Efficiency (TOWRE) of pseudoword decoding efficiency (PDE) subtest. Performance for word and non-word reading has been shown to be highly correlated (Barker et al., 1992); however, pseudoword decoding is argued to be a more reliable measure of reading development (Curtis, 1980; Gough, 1983) for early readers because unknown words are equivalent to non-words and require an online integration of orthographic and phonemic representations that is influenced less by memorized vocabulary (Seidenberg et al., 1994). Moreover, McNorgan et al. (2011) found that developmental changes in PDE were predicted by changes in the reading network over the same period, establishing the sensitivity of this measure of reading skill to developmental changes in neural dynamics. In this work, the authors noted a developmental shift in the reliance on predominantly orthographic processing (e.g., sight word recognition) to interactions between phonological and orthographic processing regions as skilled readers develop.

We quantify changes in network connectivity characteristics over time using task-related fMRI data collected at two time points from young readers (aged 8–14 and 10–17 years, respectively). We use task-related fMRI acquired while participants engage in rhyming judgments of visually presented words, which is expected to engage the neural systems that underlie orthographic and phonological processing and the interactions between these two processes. This choice, over resting state MRI (rs-MRI) reflects an interest in changes in the dynamics of neural activity related to reading, rather than general changes in brain organization. Moreover, because we are interested in network-wide changes in connectivity, we use global measures of functional connectivity rather than seed-based correlational approaches, such as psychophysical interaction (PPI) analyses that measure connectivity to and from a specific region. Interregional functional connectivity matrices are derived from cross-correlated time series drawn from task-sensitive cortical regions for concatenated runs at both time points. Graph-theoretic metrics are applied to the connectivity matrices to quantify changes in regional specialization in terms of functional segregation, which reflects specialized processing clusters within interconnected networks, and integration, a class of measures quantifying the synthesis of distributed information processing. Because reading entails the transformation of visually acquired orthographic representations into phonological representations, requiring the interaction of anatomically disparate and functionally specialized processing regions, these network measures were selected because they quantify this coordination and specialization of processing. These developmental changes in connectivity patterns that collectively represent the presence of clusters and the propensity of information transfer are then used to predict changes in reading skill, as assessed using standardized testing of pseudoword decoding at both time points (Torgesen et al., 1999). The body of literature discussed above suggests that more integrative processing within the reading network is associated with better reading performance, and this increased integrative processing is hypothesized to be reflected in changes in functional connectivity patterns. Accordingly, we use graph-theoretic connectivity measures to quantify not just the overall strength of connections within the reading network, but also the propensity of the constituent processing nodes within this network to function interdependently versus with relative independence. Longitudinal changes in these measures of functional connectivity that reflect a transition to more interdependent processing are predicted to be associated with greater improvements in reading skill.

## Materials and Methods

### Participants

Nineteen right hand dominant native English speakers age 8–14 years (10 females) at the first scanning session (T1), and age 10–17 years at the second scanning session (T2) participated from the Chicago metropolitan area in accordance with the Institutional Review Board of Northwestern University. The average interval between T1 and T2 scans was 2.5 years. Participant screening (described below) ensured that all children had average to above-average verbal and non-verbal intelligence scores. All children were reported by their parents to be free of neurological diseases or psychiatric disorders, a history of intelligence, reading, attention or oral language deficits, and were not taking medication affecting the central nervous system.

### Standardized Testing

A battery of standardized tests was administered at T1 and T2 to screen participants and establish reading skill for the analyses that follow. Participant characteristics are summarized in **Table 1**. Verbal and non-verbal (performance) IQ was assessed with the Wechsler Abbreviated Scale of Intelligence (WASI; Wechsler, 1999). Reading-related phonological processing was assessed using the elision and blending words (BW) subtests from the Comprehensive Test of Phonological Processing (CTOPP; Wagner et al., 2009), and participant skill in identifying known words was measured with the TOWRE (Torgesen et al., 1999) sight word efficiency (SW) and PDE subtests and the Woodcock-Johnson III (WJ-III; Woodcock et al., 2001) Letter-Word Identification (WID) subtest. Raw PDE scores were used as our measure of reading skill because our previous work (McNorgan et al., 2011) found this measure to be sensitive to longitudinal changes in absolute (rather than peer-relative) reading ability.

**Table 1:** Means and standard deviations for verbal IQ (VIQ), CTOPP measures elision and blending words (BW), TOWRE sight word efficiency (SW), and WJ-III letter-word identification (WID).

	Time 1	Time 2	Delta	*t*	*p*
VIQ	122.4 (15.6)	122.1 (13.0)	-0.3	-0.13	0.895
Elision	17.3 (2.6)	18.1 (1.2)	0.8	1.66	0.114
BW	15.0 (2.8)	17.2 (3.4)	2.2	3.73	0.002
SW	74.1 (10.5)	82.5 (6.8)	8.4	5.25	0.001
WID	57.8 (6.8)	64.6 (4.3)	6.8	7.6	0.001


### Rhyming Judgment Task

The rhyming judgment task was carried out in the MRI scanner, and required participants to make rhyming judgments during lexical trials for 96 word pairs (24 for each run) presented visually in the center of a screen. Words were presented for 800 ms followed by a 200 ms inter-stimulus interval (ISI). Immediately after the second word was presented, a red fixation cross was used to signal for a response during the next 2600 ms; responses outside of that time window were counted as errors. Participants were instructed to press a button with their index finger for rhyming pairs and a button with their middle finger for non-rhyming pairs using an optic response keypad. Orthography and phonology was manipulated such that word pairs matched or conflicted on one or both dimensions, resulting in a total for four conditions: in two conditions both orthography and phonology matched (O+P+, e.g., *dime–lime*) or conflicted (O-P-, e.g., *staff–gain*). Orthography matched while phonology conflicted (O+P-, e.g., *pint–mint*) in a third condition, and phonology matched while orthography conflicted (O-P+, e.g., *jazz–has*) in the fourth condition. Response time (RT) and accuracy (ACC) were recorded for each trial.

Baseline data were collected with 24 fixation trials during each run that required participants to press a button indicating the change in color (red to blue) of the fixation cross displayed on the center of a screen. The experiment included 12 trials during each run of a perceptual condition that served as a baseline for an unrelated study. For these trials, two sets of three non-alphabetic glyphs were sequentially presented. The sets of glyphs were increasing, decreasing or constant in height, and the participants indicated *via* button press whether the glyph sequences were matching (e.g., both increasing in height) or mismatching (e.g., one decreasing, the other increasing in height).

### Procedure

Following the obtainment of written informed consent and standardized testing, and in advance of the fMRI scan, participants were taught to maintain head position to minimize head movement using infrared tracker feedback in a practice mock scan. Participants also underwent a practice rhyming task in a mock scanner to ensure that they understood the task. The T1 scanning session took place within 1 week of the practice session. T1 and T2 scanning sessions occurred approximately 2½ years apart, with the precise interval used as a nuisance regressor in the analyses that follow.

### MRI Data Acquisition

Images were acquired using a 3T Siemens Trio MRI scanner with a standard 16-channel head coil at the Northwestern University Center for Translational Neuroimaging. Foam padding was used to reduce head movements. BOLD functional images were acquired in an interleaved sequence from bottom to top using echo planar imaging (EPI) with the following parameters: TE = 20 ms, flip angle = 80°, matrix size = 128 × 120, field of view = 220 mm × 206.25 mm, slice thickness = 3 mm (0.48 mm gap), number of slices = 32, and TR = 2000 ms. Scanning sessions consisted of two functional runs, each approximately 6:44 in duration. A high-resolution T1 weighted 3D structural image was acquired using the functional image orientation with the following parameters: TR = 1570 ms, TE = 3.36 ms, matrix size = 256 × 256, field of view = 240 mm, slice thickness = 1 mm, and number of slices = 160.

### Image Analysis

Imaging data were analyzed using the FreeSurfer (version 5.1.0^1^) software suite (Fischl, 2012). The anatomical volume was segmented into white matter and gray matter volumes and rendered as 3D structural surface mesh. Anatomical regions were partitioned using an automated parcellation of the gyri and sulci and mapped onto a template cortical surface mesh (Destrieux et al., 2010). Functional images were co-registered with the 3D anatomical surface for each subject and mapped onto a common structural template for group analysis with a voxel size of 3 mm^3^.

**FIGURE 1:**
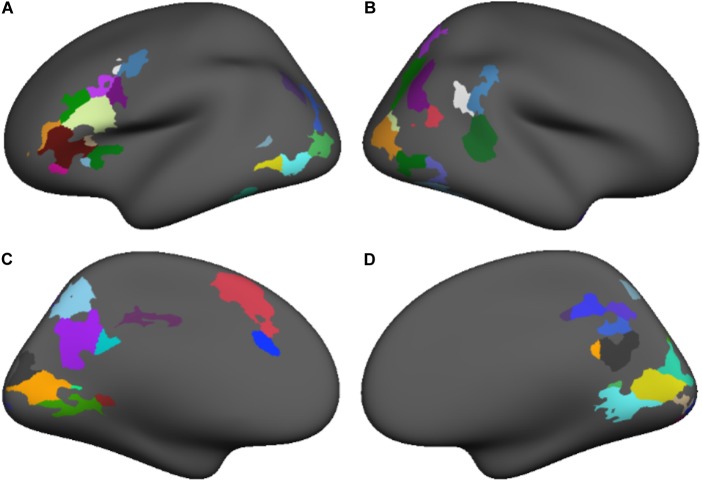
The regions demonstrating a significant task vs. baseline activation contrast at both time points subdivided based on anatomical nomenclature (coordinates shown in **Table 2**) for lateral and medial surfaces of the left **(A,C)** and right **(B,D)** hemispheres.

We conducted a subject-level general linear model (GLM) analysis of the four lexical, fixation, and perceptual conditions in the template surface space, including motion parameters as nuisance regressors. Separate GLM analyses were carried out for each study time point. A contrast between lexical and fixation conditions evaluated the main effect of the lexical conditions for each subject. Group-level random effects analyses evaluated the lexical vs. fixation contrast at each study time point across all participants. Significant activations in the group-level analyses were thresholded (*p* = 0.05) using voxel-wise false discovery rate (FDR) correction to isolate significant clusters of activation demonstrating greater lexical vs. fixation activity, as shown in **Figure 1** and **Table 2**.

**Table 2:** Coordinates for regions demonstrating a significant task vs. baseline activation contrast at both time points (shown in **Figure 1**).

	Left				Right			
		
Region	*x*	*y*	*z*	Size (mm^2^)	*x*	*y*	*z*	Size (mm^2^)
Inferior parietal gyrus	-33	-79	16	340	51	-49	25	260
	-36	-76	35	466	43	-59	16	142
					39	-70	34	750
					38	-79	13	96
Inferior temporal gyrus	-46	-47	-15	240	49	-59	-5	250
	-50	-63	-4	141				
Lateral occipital gyrus	-43	-72	-4	299	35	-83	6	528
	-35	-84	2	466	43	-74	-4	318
	-14	-96	-3	47	20	-91	-8	143
					16	-97	-7	84
Fusiform gyrus					42	-57	-13	301
Cuneus	-5	-84	18	529	6	-84	17	472
Pericalcarine gyrus	-13	-79	8	822	14	-92	-2	178
					13	-76	8	916
Lingual	-13	-64	-1	511	16	-61	1	898
Middle temporal gyrus	-51	-62	6	57				
Pars opercularis gyrus	-46	14	17	889				
	-38	21	10	66				
Pars orbitalis gyrus	-45	32	-12	96				
Pars triangularis gyrus	-42	30	3	875				
Superior parietal gyrus	-12	-75	47	45	21	-61	53	290
					31	-68	30	550
					14	-85	36	37
Caudal middle frontal gyrus	-38	2	38	39				
	-35	8	29	168				
Precentral gyrus	-50	-3	42	315				
	-45	2	26	222				
Posterior cingulate gyrus	-7	-28	39	223	7	-36	41	54
Superior frontal gyrus	-9	14	49	775				
Supramarginal gyrus					56	-40	32	565
Caudal anterior cingulate gyrus	-9	24	26					
Lateral orbitofrontal gyrus	-28	27	3	37				
Rostral middle frontal gyrus	-39	25	23	259				
	-38	36	8	161				
Precuneus	-26	-62	6	25	8	-67	50	125
	-8	-63	46	590	25	-59	7	40
	-11	-61	28	893	9	-58	32	183
					9	-45	42	267
						-61	40	172
						-59	23	536
Caudal anterior cingulate gyrus	-9	24	26	125				
Insula	-31	15	6	215				
Isthmus cingulate gyrus	-15	-47	0	77	6	-50	21	51
	-7	-49	27	131				


**FIGURE 2:**
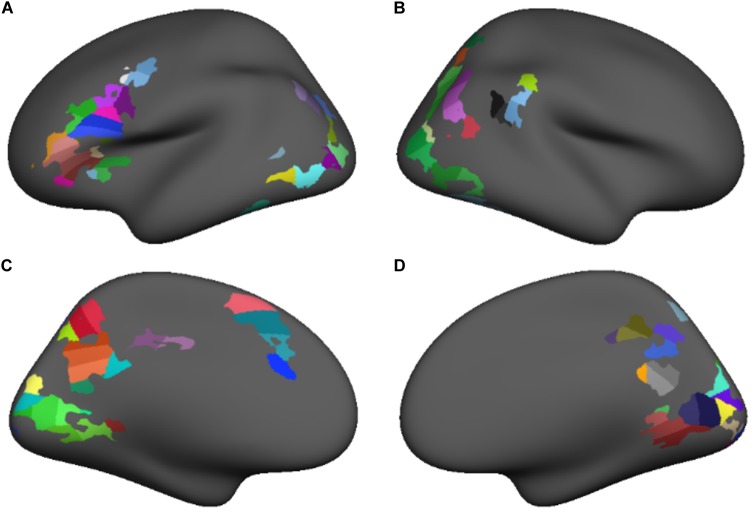
Functional regions of interest sub-parcellated into roughly equal size (approximately 400 mm^2^) for lateral and medial surfaces of the left **(A,C)** and right **(B,D)** hemispheres.

The group-level conjunctions of clusters demonstrating a significant positive lexical vs. fixation activation contrast at both time points were used to create functional regions of interest (fROIs) for the subsequent connectivity analyses. In this way, we identified a stable core orthographic decoding network – that is, regions of the cortex that were reliably more active during the lexical condition at both study time points. Notably, the clusters appearing in the intersection of the T1 and T2 contrast maps varied in size and often spanned multiple anatomical region. Because larger regions, or those spanning multiple anatomical landmarks are more likely to include functionally distinct populations, we increased distinctiveness and homogeneity among our fROIs in a two-step process. In the first step, we projected the conjunction map on the FreeSurfer template surface and overlaid the anatomical region boundaries from the Desikan-Killiany atlas (Desikan et al., 2006). Clusters spanning multiple anatomical regions were partitioned at the atlas-defined anatomical region boundaries to produce subclusters that were restricted to single anatomical regions. In the second step, we further subdivided each of the subclusters into regions of roughly equal size, using the FreeSurfer mris_divide_parcellation utility, which divides a surface-based region perpendicular to its longest axis to produce roughly equally sized subdivisions covering a specified area. All subclusters were subdivided in this way to produce 99 fROIs, each covering approximately 400 mm^2^ of cortical surface (**Figure 2**). Thus, the functionally defined reading network comprised 99 nodes of approximately equal size, were demonstrably more involved in the phonological decoding of printed words than a baseline task at both time points and were individually restricted to single anatomical regions to increase functional homogeneity within each node.

### Functional Connectivity Analysis

The BOLD time series, averaged across voxels contained within the surface space described by each functional fROI, was extracted for both functional runs at each of the T1 and T2 time points (four time series matrices in total). For each of time points T1 and T2, the Run 1 and Run 2 time series were concatenated to yield a single time series for each of T1 and T2. For each time point T1 and T2, inter-regional functional connectivity was estimated by the pairwise zero-lag cross-correlations between each fROI to produce two 99 × 99 connectivity matrices, **M**, where each entry **M**_ij_ provides the estimated functional connectivity between regions *i* and *j*. We computed common measures of functional integration and segregation within the functional connectivity matrices at Time 1 and Time 2 using the Brain Connectivity Toolbox (BCT; Rubinov and Sporns, 2010).

**FIGURE 3:**
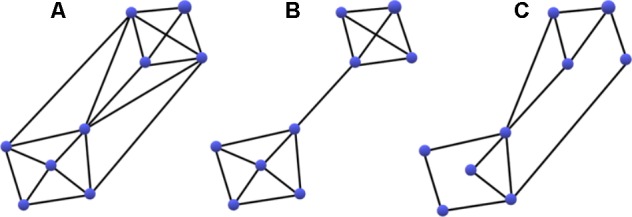
Demonstration of an increase in Modularity **(B)** and a decrease in Transitivity **(C)** compared to a densely integrated network **(A)**. Transitivity measures the fraction of a node’s neighboring connections that are also connected with each other to form cliques. Modularity is an indicator of the degree to which the network may be subdivided into clearly delineated and non-overlapping groups. Note, both changes may result in more efficient processing.

Functional integration reflects a networks capacity for information transfer and was evaluated using a measure of *Global Efficiency* (GE) and *Diameter*. GE represents the reciprocal of the path length between nodes, with higher GE indicative of a network comprised of clusters connected *via* shorter paths allowing for more efficient processing. Diameter indicates the longest path between any two nodes after computing the shortest path between any two nodes, which reflects the breadth of the network. Functional segregation was measured by *Transitivity* and *Modularity*. Functional segregation, as the name implies, is the division of a larger network into smaller functionally specialized sub-networks. Functional subnetworks are evident among a cluster of nodes with activity that is less correlated with that of the rest of the network, suggesting that those nodes are engaged in a different processing task (Fornito et al., 2012). Transitivity measures the fraction of a node’s neighboring connections that are also connected with each other to form cliques. A high degree of transitivity is indicative of a dense interconnected network, or densely connected clusters within a network, whereas a low degree of transitivity is indicative of a sparser network organization with less functional specialization. Modularity is an indicator of the degree to which the network may be subdivided into clearly delineated and non-overlapping groups. A higher measure of modularity is indicative of a more clustered network organization. These concepts are illustrated in **Figure 3**, which demonstrates extreme examples of changes in transitivity and modularity. Additional information about each of these measures can be found in Table A1 in Rubinov and Sporns (2010).

Changes in functional connectivity between the two study time points within each participant were measured by the difference (Δ) between connectivity measures at each scanning session (T2 - T1). Likewise, differences in measures of reading skill at each time point were calculated to assess changes in reading skill over the same time period. A linear regression model assessed whether changes in measures of functional integration or segregation accounted for changes in measures of reading skill.

## Results

### Behavioral Analysis

Mean and standard deviations for RT and accuracy at T1 and T2, and the change in measure of reading skill (PDE) for lexical trials are presented in **Table 3**. Measures of ΔSW and ΔPDE were significantly and positively correlated (*r* = 0.70, *p* = 0.001; uncorrected), consistent with their mutual dependence on orthographic and phonologic integration and indicating that these skills were developing in parallel in our sample. PDE scores ranged from 19 to 63 (out of a possible 63) for T1 and from 31 to 59 for T2.

**Table 3:** Mean and standard deviations at T1 and T2 for reaction time (RT) and accuracy (ACC) for the lexical trials, the change in measure of reading skill, pseudoword decoding efficiency (PDE), and change in transitivity.

	Time 1	Time 2	Delta	*t*	*p*
RT	1285 (359)	1231 (339)	-54	-1.74	0.099
ACC	83.1% (8.6%)	80.5% (9.0%)	-2.6%	-1.81	0.087
PDE	40.2 (11.9)	46.9 (7.7)	6.7	3.57	0.002
Transitivity	0.36 (0.05)	0.37 (0.05)	0.01	0.95	0.356


*PDE performance was significantly improved at T2 compared to T1. There was no significant change in transitivity between T1 and T2.*

### General Linear Model Analysis

**FIGURE 4:**
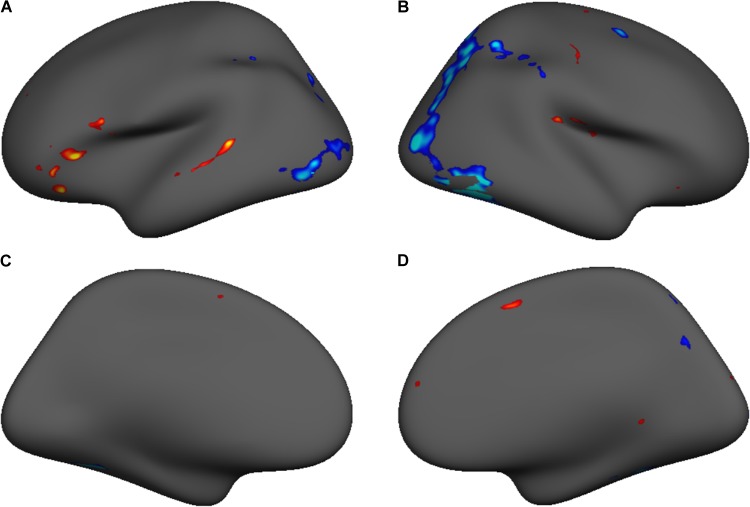
Group level contrast map showing significant voxel-wise FDR corrected (*p* = 0.05) T1 vs. T2 differences in the Lexical vs. Fixation contrast for lateral and medial surfaces of the left **(A,C)** and right **(B,D)** hemispheres.

Though the evaluation of developmental changes in regional activity during single-word reading was not a central goal of this study, cortical surface contrast maps were generated to identify the reading network at T1 and T2. This afforded the opportunity to compare the activation maps within participants to identify developmental changes in fMRI activity for this task. We performed a random-effects within-subjects *t*-test of T1 vs. T2 in the Lexical vs. Fixation contrast. **Figure 4** shows a group level contrast map of cluster-size corrected significant activation differences in this contrast, within a per-voxel significance threshold of *p* < 0.001 (FWE). Peak voxel statistics are presented in **Table 4**. Overall, T1 was associated with more activation in left hemisphere phonological processing areas (STG; IFG), and T2 was associated with more activation in orthographic processing areas (FG).

**Table 4:** Coordinates for size-corrected clusters (*p* = 0.001) demonstrating significant T1 vs. T2 task-related activation (shown in **Figure 4**).

	Left				Right			
		
Region	*x*	*y*	*z*	Size (mm^2^)	*x*	*y*	*z*	Size (mm^2^)
Inferior parietal gyrus					31	-63	41	1029
Inferior temporal gyrus					44	-56	-11	380
					55	-58	-8	311
Lateral occipital gyrus	-41	-77	-2	655	28	-83	6	455
Fusiform gyrus	-30	-63	-15	102				
Superior temporal sulcus	-58	-40	6	181				
Pars opercularis gyrus	-48	13	18	98				
Pars triangularis gyrus	-36	29	4	156				
Superior parietal gyrus					30	-50	50	175
Lateral orbitofrontal gyrus	-35	30	-15	97				


### Functional Connectivity Analysis

**FIGURE 5:**
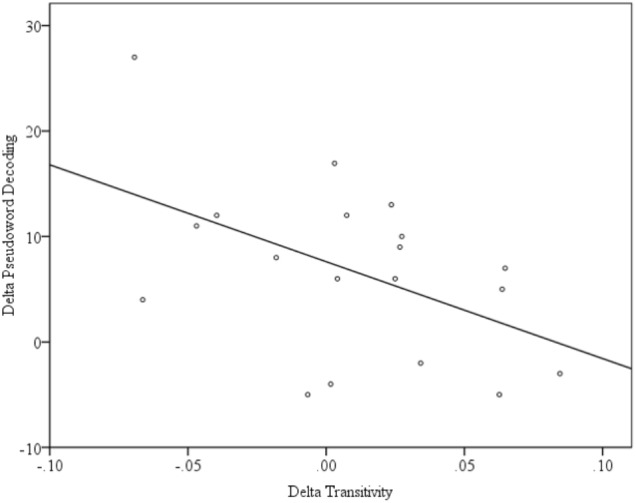
Scatterplot of the significant relationship between the change in Transitivity and PDE.

An initial description of the relationship between connectivity and reading skill was obtained from uncorrected zero-order Pearson correlations between our measure of reading skill and each of the network metrics. Transitivity at T1 was not significantly correlated with PDE at T1 (*r* = 0.07, *p* = 0.79), but Transitivity at T2 is correlated with PDE at T2 (*r* = 0.48, *p* = 0.04). Because reading scores increased over this same period, there was an overall pattern wherein reading scores increased and correlations between transitivity and reading scores increased within the same period. Pearson correlations calculated between T2 – T1 changes (Δ) in functional connectivity (ΔGE, ΔDiameter, ΔTransitivity, and ΔModularity) and reading skill (ΔPDE) indicated a significant relationship between the change in Transitivity and PDE (*r* = -0.49, *p* = -0.03; uncorrected), as shown in **Figure 5**. Because transitivity is an index of functional segregation, this indicates that an increase in reading skill is accompanied by a reduction in the proportion of functionally distinct clusters of activity. In short, the children who made the greatest gains in reading skill were those for whom the network made the largest transition from a collection of relatively independently functioning nodes to a coherent (i.e., unified) network.

We more rigorously assessed these relationships controlling for nuisance variables, including age, verbal ability (VIQ), phonological ability (Elision, BW), general letter and word recognition ability (WID, SW), and task performance (RT, ACC), at T1 and T2. Partial correlations between Transitivity and PDE at T1 (*r* = -0.41, *p* = 0.209) and at T2 (*r* = 0.10, *p* = 0.771) were non-significant, indicating that the significant zero-order correlation at T2 was largely driven by variables of non-interest. Our reanalysis of the relationships among developmental changes in connectivity and reading ability employed a stepwise linear regression that additionally controlled for nuisance variables, including age at enrollment, and T2 - T1 changes in verbal ability (ΔVIQ), age (ΔAge), phonological ability (ΔElision, ΔBW), general letter and word recognition ability (ΔWID, ΔSW), and changes in task performance (ΔRT, ΔACC), *F*(1, 17) = 15.92, *R*^2^ = 0.48, Adj. *R*^2^ = 0.45, *p* < 0.01, prior to entering all four functional connectivity measures into a model predicting changes in reading skill (ΔPDE). The final model includes ΔSW ($\eta_{p}^{2}$ = 0.72,p = 0.001) and ΔTransitivity ($\eta_{p}^{2}$ = -0.55,p = 0.019) as significant predictors of ΔPDE, *F*(2, 16) = 14.14, *R*^2^ = 0.64, Adj. *R*^2^ = 0.59, Δ*R*^2^ = 0.16, *p* < 0.001. No other variables were significant predictors of changes in PDE. These results indicate that, apart from changes in sight word reading, with which PDE has long been known to be highly correlated (Barker et al., 1992), changes in our measure of reading skill were predicted only by transitivity changes within the reading network.

Though we took measures to restrict our analyses to brain regions that were preferentially engaged in reading at both time points, the connectivity analyses described above were performed on functional connectivity estimates computed from two intact concatenated time series. Because the experiment used a fast event-related design, both time series contained intermittent periods (e.g., the perceptual and fixation trials) during which participants were not actively engaged in reading. This introduces the possibility that the connectivity metrics within the functionally defined reading network may have been influenced by time series correlations associated with non-reading activity. Given the design of the experiment, it is impossible to isolate segments of the intact time series that can be confidently ascribed to reading or non-reading processing. Rather, the best available means of addressing this concern was by first modeling the fixation and perceptual trials with the GLM, and saving the residualized values as our best estimate of what the time series would look like had these conditions been excluded from the design. We then computed connectivity statistics on functional connectivity estimates derived from the residualized time series. When we do so, we find that ΔTransitivity ($\eta_{p}^{2}$ = - 0.40,p = 0.09) remained a marginally significant predictor of ΔPDE.

## Discussion

We hypothesized that changes in reading skill should be reflected in changes in the dynamics of processing in the reading network, specifically that changes indicative of more efficient processing will predict improvements in reading performance. We explored this hypothesis by examining longitudinal changes in neural connectivity within a functionally defined reading network in young readers. This research provides evidence that improvements in reading skill over time are predicted by the nature and degree of changes among connectivity patterns within the reading network. This is consistent with broader theories of neural development that stipulate cognitive performance improves as a result of optimized connectivity throughout the lifespan (Casey et al., 2005; Bassett et al., 2009), and connectionist models of reading development that propose a shift in interregional dependency within the reading network (Koyama et al., 2011; Liu et al., 2018).

Lending critical support to our hypothesis, the hierarchical model additionally found that changes in functional segregation, specifically transitivity, predicted changes in pseudoword decoding. Transitivity is an index of functional segregation, with large transitivity values indicating that a network contains many embedded cliques, each working somewhat independently of each other. Thus, a decrease in transitivity indicates a developmental decrease in the proportion of such clusters. Stated conversely, a reduction in transitivity reflects an overall increase in processing coherence among these regions, such that the nodes within the reading network work less independently and more cooperatively. Because a decrease in transitivity predicted an improvement in reading skill, this indicates that the unification of the reading network through cooperative processing is a critical driver of gaining reading skill. Transitivity has been identified as an important component of neural connectivity (Zalesky et al., 2012; Baronchelli et al., 2013; Goñi et al., 2014) and used in previous work to distinguish dysfunction from normal processing, such as in Schizophrenia (Anderson and Cohen, 2013) and Alzheimer’s (Stam et al., 2006); however, this work appears to be the first to apply transitivity to task-related behavior.

We note here an apparent inconsistency between the positive zero-order correlation between PDE and Transitivity at Time 2, and the significant zero-order correlation between the change in PDE and the change in Transitivity between Time 1 and Time 2. We find that the zero-order correlations between PDE and Transitivity at these time points were confounded by several variables with obvious or non-interesting relationships with PDE, and that when these variables are accounted for, Transitivity is not significantly correlated with PDE at either time point. This lack of relationship indicates that, at some arbitrary baseline point in reading development, a child at a particular level of orthographic decoding skill could demonstrate either relatively high or relatively low Transitivity within the reading network. Importantly, the negative relationship between the change in transitivity and PDE remains significant, even when these confounding variables are accounted for. This suggests that, regardless of baseline reading network Transitivity, a decrease in Transitivity across time within this network is important for ongoing reading development, as it marks its ongoing reorganization.

Reading entails coordinated contributions of multiple functionally specialized brain regions that process orthographic, phonological, and semantic information. As such, most theories of reading development characterize improvements in reading as the result of changes in the efficiency by which distributed components of the reading network interact. Recent literature incorporating DTI measures in a machine learning application to predict reading skill demonstrates that connectivity within the putative reading network is critical for normal reading development (Cui et al., 2016). Whole brain connectivity estimated from rhyming task data indicates decreased connectivity in the visual word form area (VWFA) and regions associated with visual processing, and increased connectivity among temporal regions for dyslexics compared to normal readers (Finn et al., 2014), suggesting that dyslexics depend on a less developed reading mechanism reliant on phonological processing. Moreover, early connectivity changes have been shown to precede and predict the location of the development of a VWFA (Saygin et al., 2016). The VWFA appears specifically tuned to the orthography of a reader’s familiar language and is considered an important developmental milestone for reading fluency. The changes in functional connectivity patterns presented here may reflect the refinement of the VWFA that emerges as a product of the increased interaction among regions in the reading network. Price and Devlin (2011) suggest an increased responsiveness to orthography is driven by the development of integrated bottom-up sensory processing and top-down phonological and semantic processing regions, such that interaction between orthographic, phonological, and semantic information becomes increasingly automatized. This account is consistent with most accounts of reading development and more general explanations of cognition, whereby development is the product of an integrated bottom-up and top-down system.

Resting-state literature has been used to argue for a domain general role of the purported reading network (Vogel et al., 2013). Koyama et al. (2011) has shown that increased resting state connectivity between language processing regions and motor regions in both adults and children predicts reading skill, which they suggest reflects more automated processing. Our findings neither support nor refute the domain-general hypothesis; however, they provide an interesting context to interpret the resting state literature within Price and Devlin (2011) Interactive Account framework. The Interactive Account argues that functionally specialized modules within the reading network, such as the putative VWFA, are a consequence of the interactions between regions within this subnetwork. Resting-state data shows more connectivity between temporal regions and VWFA for adults compared to children indicating that development of the reading network entails increased functional interconnectivity. Connectivity within this network appears to also be related to reading impairment: resting state magnetoencephalography (MEG) has provided evidence that reading impaired children have altered global and localized temporoparietal connectivity believed to reflect less efficient signal processing (Dimitriadis et al., 2013), and less uniform temporal and spatial patterns attributed to altered information exchange in comparison to non-impaired readers (Dimitriadis et al., 2016). Improvements in reading following intervention for normal children and children with reading difficulties has been correlated with increased interactivity between core networks in reading and cognitive control (Horowitz-Kraus et al., 2015). Finally, González et al. (2016) show decreased diameter and increased eccentricity, characteristic of less efficient connectivity, in electroencephalogram (EEG) resting state data for dyslexic readers compared to normal controls, and suggest deficits observed in dyslexics are due to altered network connectivity rather than specific regional dysfunction. The resting-state connectivity literature collectively points to a model in which measures of connectivity within the core reading network, and between the reading network and other domain general areas can be used to differentiate readers at different skill levels. Whether functional connectivity is computed from task or resting-state data, it measures moment-to-moment processing dynamics within the brain. If functional specialization is an emergent property of these processing dynamics, it follows that reading network development will be intimately tied to whole-brain functional connectivity patterns that dictate the efficiency with which phonological and orthographic representations may be transformed and transmitted to support reading.

### Limitations and Future Directions

As noted earlier, one potential concern was that connectivity within the intact time series was estimated in part from non-lexical task activity. Because a fast event-related design was used, one cannot easily disentangle lexical and non-lexical processing. To address this potential confound, we used the approach of regressing out non-lexical activity, applying the logic on which analyses of covariance (ANCOVA) are based. It is important to note, however, that one should not construe the residualized time series as reflecting reading-only processing. The general linear approach models an idealized hemodynamic response function (HRF) that is convolved with a vector of event onset times. This approach is based on an assumption that the single HRF describes the response characteristics of each voxel equally well. We know, however, that the HRF varies regionally (Lu et al., 2006). This is seldom a concern for conventional contrast-based approaches because all comparisons are performed within-voxel, and thus, the spatial inhomogeneity of the HRF is equated across conditions. However, because the non-lexical activity estimate will be non-uniformly accurate, functional connectivity estimates of reading-related brain activity will be confounded by inter-regional differences in the degree to which the canonical HRF accurately models non-reading brain activity. Because connectivity estimates directly depend on inter-regional activity correspondence, this non-uniformity would be expected to influence connectivity metrics. The fast event-related design used in this experiment thus limits the confidence with which we can argue that our connectivity measures reflect purely reading-related functional connectivity. However, we found that changes in network transitivity predicted changes in reading skill on residualized lexical event time series when non-lexical events were regressed out. In conjunction with the rigorous data-driven approach to defining the network of interest, this suggests that the degree to which the constituent processing regions within the reading network function in tandem is relevant to the improvement of reading skill.

The whole network connectivity measures that we compute numerically summarize characteristic connectivity patterns among all nodes within the network. They cannot, however, be decomposed to make claims about specific nodes or connections. For example, though the reading network is largely left-lateralized, our functional network included nodes in the right hemisphere. Increased transitivity may reflect increased interhemispheric communication in some individuals, and increased posterior–anterior communication in others. The global network transitivity measure does not distinguish between the two, but this is not consequential to the main point that, regardless of the dominant directionality of communication, increased cooperative activity among nodes is associated with improved reading ability. Likewise, our global network measure does not speak to connectivity motifs outside the reading network. It is thus possible that changes in other networks, such as the default mode networks identified in resting state connectivity studies are additively predictive of reading skill as attention and memory skills continue to develop. However, we take the position that skilled reading depends on multisensory interactions between brain regions that support orthographic and phonological processing, and that these interactions are supported by the skill-dependent connectivity between these regions. Our findings are consistent with this perspective, and should be seen as providing a big picture perspective that complements other network-level studies using seed-based approaches (e.g., dynamic causal modeling; Cao et al., 2008) or focusing on specific anatomical connectivity tracts (e.g., Gullick and Booth, 2014). Collectively, this body of literature suggests that skilled reading requires the coordinated effort of a network of brain regions, each contributing uniquely and importantly to the overall task of decoding between phonographic and orthographic representations.

Here, we propose that differences in developmental trajectories of reading network connectivity may function as a mechanism for explaining individual differences in reading ability. These findings, along with McNorgan and Booth (2015) and McNorgan et al. (2013), suggest that learning to read involves promoting interactions among these nominally modality-specific processing areas to make them work more cooperatively. It remains unclear how cooperative processing across these regions underlies reading development. Segmentation of the functional network reported here into occipito-temporal and parietal nodes may allow for investigation into whether there is a developmental change in connectivity that corresponds to a shift from phonological to orthographic processing. Additionally, further elucidation of reading network processing dynamics might involve incorporating methodology with faster time resolution (e.g., near-infrared spectroscopy or event-related potentials) to identify the sequence of interactions among these regions.

### Summary

Our longitudinal analysis examined the predictive relationship of changes in graph-theoretic metrics of task-related functional connectivity within fMRI data collected from a sample of typically developing children over a period of several years. Our analysis found that improvements in reading skill were predicted by a desegregation of nominally functionally specialized regions within the putative reading network. These findings provide additional insight into how developmental changes within reading network connectivity contribute to gains in reading skill in typically developing readers and suggest a model in which typical reading development entails increased interdependence among brain regions that typically support auditory and visual processing across other contexts.

## Ethics Statement

This study was carried out in accordance with the recommendations of the Institutional Review Board of Northwestern University. The protocol was approved by the Institutional Review Board of Northwestern University. Parents of all subjects gave written informed consent in accordance with the Declaration of Helsinki.

## Data Availability

The data for this study will be made available upon request.

## Author Contributions

GS, JB, and CM contributed conception and design of the study. JB oversaw data collection. GS performed the statistical analysis and wrote the first draft of the manuscript. CM and GS wrote sections of the manuscript. All authors contributed to manuscript revision, and read and approved the submitted version.

## Conflict of Interest Statement

The authors declare that the research was conducted in the absence of any commercial or financial relationships that could be construed as a potential conflict of interest.

## Footnotes

1 http://surfer.nmr.mgh.harvard.edu
